# Defects in Innate and Intrinsic Immunity in Morocco: A Retrospective Analysis of the Genetic Landscape and Clinical Correlations

**DOI:** 10.20411/pai.v10i2.845

**Published:** 2025-11-03

**Authors:** Marwa Refaat, Chaymae Oujane, Abderrahmane Errami, Zahra Aadam, Abderrahmane Moundir, Bouchra Baghad, Sanae Zaidi, Halima Kholaiq, Assiya El Kettani, Ibtihal Benhsaien, Fatima Ailal, Asmaa Drissi Bourhanbour, Jalila El Bakkouri, Ahmed Aziz Bousfiha

**Affiliations:** 1 Laboratory of Clinical Immunology, Infection and Autoimmunity (LICIA), Faculty of Medicine and Pharmacy Casablanca, Hassan II University, Casablanca, Morocco; 2 Department of Infectious Diseases and Pediatric Clinical Immunology, Mother-Child Hospital Abderrahim Harouchi, CHU Ibn Rochd, Hassan II University, Casablanca, Morocco; 3 Immuno-Serology Laboratory, CHU Ibn Rochd, Casablanca, Morocco; 4 Department of Dermatology and Venerology, CHU Ibn Rochd, Hassan II University, Casablanca, Morocco; 5 Laboratory of Immunopathology-Immunomonitoring-Immunotherapy, Mohammed VI University of Health Sciences (UM6SS), Casablanca, Morocco

**Keywords:** Innate Immune Deficiencies, Genetic Variations, Pathogens, Susceptibility, Primary Immunodeficiencies, PID, Inborn Errors of Immunity, IEI

## Abstract

**Background:**

Susceptibility to common infectious diseases is often linked to innate immune deficiencies. Patients may present normal standard immunological profiles but remain highly vulnerable to infections, complicating diagnosis. This study investigates innate and intrinsic immune deficiencies and their genetic underpinnings in Moroccan patients, emphasizing early detection and personalized care.

**Methods:**

A retrospective analysis was conducted using data from the Moroccan Inborn Errors of Immunity (IEI) registry (2008–2024). Included were patients with confirmed innate or intrinsic immunodeficiencies based on CBC, CRP, immunoglobulin levels, lymphocyte subpopulations, and whole-exome sequencing. Classification followed the 2022 IUIS criteria.

**Results:**

Among 884 patients with IEI, 79 (∼9%) had innate or intrinsic immunodeficiencies, with genetic confirmation in 46 (58%). Of these, 23 (50%) were diagnosed with Mendelian susceptibility to mycobacterial disease (MSMD), involving mutations in the *IL12RB1*, *STAT1*, *IFNGR1*, *SPPL2A*, *TYK2*, and *TBX21* (*T-bet*) genes. Chronic mucocutaneous candidiasis (CMC) was found in 15 (32%) patients, linked to *STAT1* and *IL17RA* mutations. Severe viral infection predisposition was seen in 3 patients (*POLR3A, IFIH1, TLR7XL*) and bacterial susceptibility in 3 others (*IRF4, IFNGR1, NCSTN*). Novel variants were identified, including *IRAK4* c.277delT (p.F93fsX26), not previously reported, and *SNORA31* (n.36T>C), previously seen in Saudi Arabia, now found in a Moroccan case of herpes simplex encephalitis.

**Conclusion:**

This study reveals the genetic complexity of innate immune disorders in Morocco, with a notable prevalence of MSMD and CMC. It underscores the value of early genetic screening to guide diagnosis and improve patient outcomes.

## INTRODUCTION

The innate immune system is the first line of defense against bacterial and viral pathogens. It detects pathogens through specialized receptors called pathogen recognition receptors (PRRs), which identify pathogen-associated molecular patterns (PAMPs) and danger-associated molecular patterns (DAMPs). Upon recognition, these receptors trigger a defense response by activating the production of chemokines and cytokines, which help coordinate the immune system's attack against invading pathogens. This rapid, nonspecific response is important for early infection control [1–4].

Innate and intrinsic immunity disorders are a subset of inborn errors of immunity (IEI) characterized by monogenic abnormalities that impact the number or function of cellular components involved in innate immunity, excluding phagocyte or complement defects [4–7]. This group is classified in Table 6 of the International Union of Immunological Societies (IUIS) classification system [8].

The incidence of IEI disorders varies widely, ranging from 1 in 500 to 1 in 1,000,000 depending on the specific genetic defect and geographical region [9]. In the Middle East and North Africa region, 570 patients with innate immune defects are reported across 13 countries [10], although this number is likely underestimated due to missed diagnoses.

Defects in intrinsic and innate immunity lead to susceptibility to a limited range of common pathogens, including Mendelian susceptibility to mycobacterial disease (MSMD), Epidermodysplasia verruciformis (associated with human papillomavirus), predisposition to severe viral infections (such as influenza and COVID-19), herpes simplex encephalitis (HSE), and susceptibility to certain parasitic, fungal, and invasive bacterial infections [8, 11]. Due to this specific susceptibility to common pathogens, many specialists often do not investigate primary immunodeficiency in the presence of these infections. Furthermore, traditional immunological tests, such as lymphocyte counts, immunoglobulin levels, and vaccine responses, often appear normal, complicating the diagnosis of intrinsic and innate immune defects [12]. Therefore, this study aims to determine the position of innate and intrinsic immunity disorders within the spectrum of IEI and to identify the types of susceptibilities related to them in Morocco in order to raise awareness among physicians about these disorders. Early genetic diagnosis of innate immune deficiencies will help improve quality of life for patients by decreasing mortality rates and complications, including resistance to treatment and the associated unusual causative microorganisms.

## METHODS

A retrospective analysis of patients with innate and intrinsic immunodeficiencies from the Moroccan IEI registry from 2008 to 2024 has been performed. Cases of innate and intrinsic immune deficiencies were identified through the Moroccan IEI registry, which receives referrals from pediatricians, infectious disease specialists, dermatologists, and other clinicians across the country. Patients typically present with recurrent, severe, or unusual infections, for example, mycobacterial disease, chronic mucocutaneous candidiasis (CMC), HSE, or with non-infectious manifestations such as lymphadenopathy, autoimmune features, or dermatological abnormalities. In addition, initial suspicion was raised when patients showed infections with common pathogens but with unusually severe or persistent clinical courses, particularly when standard immunological work-up (ie, lymphocyte counts, immunoglobulin levels, vaccine responses) appeared normal. Diagnosis was confirmed by genetic testing (ie, mainly whole exome sequencing), and classification followed the IUIS 2022 criteria. Functional tests, carried out both in our laboratory and through international collaborations, including interleukin-12 (IL-12) and interferon (IFN)-γ assays and bioinformatic tools, have clarified how these variants impair immune signaling.

The study included patients whose diagnoses were supported by clinical features and confirmed through laboratory assessments (ie, CBC, CRP, immunoglobulin levels, lymphocyte subpopulation analysis) and whole exome sequencing.

Patients with genetically confirmed innate immunodeficiency and those presenting with clinical or biological symptoms suggestive of the condition were included. Patients with human immunodeficiency virus (HIV) and with autoimmune disease, complement deficiency, and other IEI were excluded. The Moroccan IEI registry is maintained at the Infectious Diseases and Pediatric Clinical Immunology Department, Mother-Child Hospital, Abderrahim HAROUCHI, CHU Ibn Rochd.

No human research studies were performed. Thus, no approval from appropriate institutional review boards or human research ethics committees was required to undertake the preparation of this report.

## RESULTS

### Epidemiologic Characteristics of Defects in Intrinsic and Innate Immunity in Moroccan Patients

Of 884 patients diagnosed with IEI, 79 (∼9%) were diagnosed with innate and intrinsic immunodeficiencies, and 46 (58%) received a genetic diagnosis. Among the latter, 23 (50%) were diagnosed with MSMD due to genetic variations in the *IL12RB1, STAT1, IFNGR1, SPPL2A, TYK2,* and *TBX21* (T-bet) genes. CMC was identified in 15 (32%) patients and was associated with variations in the *STAT1* and *IL17RA* genes. Additionally, 3 cases of severe viral infection predisposition were linked to a *POLR3A, IFIH1*, and *TLR7XL* variation, 3 cases of bacterial infection predisposition were linked to an *IRF4, IFNGR1,* and *NCSTN* variation, 1 case of HSE was linked with a *SNORA31* variation, and 1 case was linked to an *IRAK4* deficiency. In our cohort, parental consanguinity was present in 51.1% of cases. The diagnostic delay has a median and IQR of 35 (6-80), reflecting the lack of awareness of emerging diseases unfamiliar to clinicians and the lack of diagnostic facilities due to the high cost of required molecular tests (Table 1).

**Table 1.** Epidemiologic Characteristics of Defects in Intrinsic and Innate Immunity Patients

Category	No. of cases	M/F Ratio	Consanguinity (%)	Age of onset median + IQR “MO”	Age of diagnosis “median + IQR “MO”	Delay in diagnosis median + IQR “MO”
MSMD	23	12/11	65%	6 (3-51)	48 (33-152)	35 (6-80)
CMC	15	7/8	37%	36 (8-180)	96 (60-168)	31 (5-59)
Predisposition to severe viral infection	3	3/0	non	-	-	-
Predisposition to bacterial infection	3	2/1	33%	-	-	-
HSE	1	M	No	0 “ after birth”	36	36
*IRAK4* deficiency	1	M	Yes	1	48	47

M/F ratio: male/female ratio. MO: month. MSMD: Mendelian Susceptibility to Mycobacterial Disease. CMC: Chronic Mucocutaneous Candidiasis. HSE: Herpes Simplex encephalitis.

### Common Clinical Manifestations

Patients with defects in intrinsic and innate immunity presented with various clinical manifestations during diagnosis (Table 2). A previous history of recurrent infection was reported in 16 of 23 patients with MSMD (70%) and in all 15 patients with CMC (100%).

**Table 2.** Common Clinical Manifestations of Moroccan Patients with Defects in Intrinsic and Innate Immunity

Category	MSMD	CMC	Predisposition to severe viral infection	Predisposition to bacterial infection	HSE	*IRAK4* deficiency
Infectious diseases	16	15	1	-	-	1
Lymphatic and hematologic disorders	13	3	-	-	-	1
Pulmonary and respiratory disorders	7	4	-	2	-	-
Dermatological conditions	1	11	-	1	-	-
Gastrointestinal and abdominal conditions	3	3	-	-	-	-
Musculoskeletal disorders	3	1	-	-	-	-
Cardiovascular disorders	1	-	-	-	-	-
Central nervous system disorders	3	1	1	-	1	-
General symptoms and signs	4	-	-	-	-	-
Oral and dental conditions	-	14	-	-	-	-
Additional specific conditions	2	2	1	-	-	-

In patients with MSMD, the second common complication was lymphatic and hematologic disorders (n=13; 57%), which include left axillary lymphadenopathy, bilateral necrotic cervical lymphadenopathy with fistula, deep abdominal lymphadenopathy, splenomegaly and hepatosplenomegaly, anemia, and recurrent pancytopenia. The third common complication was pulmonary and respiratory disorders in patients with MSMD (n=7; 30%), which included pericarditis, pulmonary infiltrates, diffuse interstitial lung disease with apical reticular changes, symmetrical arthralgia of the lower extremities, laryngeal granuloma, chest wall inflammatory swelling, constrictive pericarditis, and asthma.

Among patients with CMC, the second common complication was oral and dental conditions (n=14; 37.5%), which include oral candidiasis, oral squamous cell carcinoma, oral thrush, gingivostomatitis with a thrush bulb, labial edema, nail hypoplasia, oral ulceration, thrush buccal relapses, and gingivitis. The third common complication was dermatologic conditions (n=11; 58%), which include warts of the hands and feet, recurrent eczema, intertrigo and diarrhea since childhood, onychomycosis, scalp alopecia, facial erythema, periorbital eczema, papular lesions of the face and nose, crusted hyperkeratotic erythematous lesions, centro facial papulopustular lesions, blepharitis, angular-cheilosis, atrophic glossitis, gingivostomatitis with a thrush bulb, labial edema, nail hypoplasia, and eczematiform lesion.

In patients with MSMD, 39% reported left axillary lymphadenopathy, 13% had proven tuberculosis, 13% reported salmonellosis, 13% reported bilateral necrotic cervical lymphadenopathy with fistula, and 13% reported asthma.

In patients with CMC, 47% reported oral candidiasis, 26% reported oral thrush, 26% reported scalp alopecia, 26% reported dermatological manifestations, 21% reported onychomycosis, and 21% reported recurrent fungal infections (Table 3).

**Table 3.** Common Manifestations Seen in Patients with MSMD and CMC

**Common manifestations of MSMD**	**No. of diseases (%)**
Left axillary lymphadenopathy	39%
Salmonellosis	13%
Bilateral necrotic cervical lymphadenopathy with fistula	13%
Asthma	13%
**Common manifestations of CMC**	**No. of diseases (%)**
Oral candidiasis	39%
Oral thrush	26%
Scalp alopecia	26%
Skin infections and manifestations	26%
Onychomycosis	21%
Recurrent fungal infections	21%

The patient with *IRAK4* deficiency suffers from inguinal adenitis, pneumococcal meningitis, multiple hepatic abscesses, a soft tissue abscess in the thorax, the appearance of recurrent ringworm of the scalp, and cervical lymphadenopathy.

The patient with HSE was born with bilateral blindness and was hospitalized for febrile meningitis.

### Immunological Findings

Table 4 shows the spectrum of immunological findings in the study population. The total count of lymphocyte subsets, including CD3^+^, CD4^+^, CD8^+^, CD19^+^, and natural killer cells, was within the normal range in almost all patients. Most patients with defects in intrinsic and innate immunity also had a normal range of immunoglobulin (Ig)A, IgG, IgM, and IgE. Some patients suffering from MSMD and CMC showed slightly high IgG, IgM, and IgA serum levels. A patient with *IRAK4* deficiency showed hyper-IgE.

**Table 4.** Immunological Findings of Moroccan Patients with Defects in Intrinsic and Innate Immunity

Category	MSMD Mean	CMC Mean	Predisposition to severe viral infection	Predisposition to bacterial infection	HSE	*IRAK4* deficiency
IgA g/L	0.97 (0.1-0.86)	2.6 (0.45-7.2)	Normal	-	Normal	Normal
IgG g/L	15.6 (2.4-12.3)	14.6 (5.5-23.3)	Normal	-	Normal	Normal
IgM g/L	1.97 (0.251.62)	4.19 (0.85-3.9)	Normal	-	Normal	Normal
IgE IU/mL	53.5 (inf-200)	311.9 (0.67-1150)	Normal	-	Normal	Hyper IgE 8000IU/L
CD3/mm^3^	4552 (1157-9921)	1865 (1200-2414)	Normal	-	Normal	3301 C/mm^3^
CD4/mm^3^	2397 (520-4785)	959.4 (600-2000)	Normal	-	Normal	1906 C/mm^3^
CD8/mm^3^	1918 (399-4940)	665 (434-1217)	Normal	-	Normal	1209 C/mm^3^
CD19/mm^3^	1798 (330-4071)	527 (230-1023)	Normal	-	Normal	744 C/mm^3^
NK	619 (260-913)	65.5 (8-150)	Normal	-	Normal	Normal

### Genetic Analysis

Our study included 23 MSMD patients (12 male and 11 female). The mean age of patients was 4 years (1–17 years). The mean age at onset was 47 months, the mean age at diagnosis was 87 months, and 65% were born to consanguineous parents (Table 1). Twelve variations have been identified in 6 genes: *IL12RB1* in 9 patients, *STAT1* in 7 patients, *SPPL2A* in 2 patients, *IFNGR1* in 2 patients, *TYK2* in 2 patients, and *TBX21* in 1 patient. Variations identified in the *STAT1* gene were associated with autosomal dominant (AD) inheritance, and all other identified variations were associated with autosomal recessive (AR) inheritance. Six patients had the same homozygous variation, c.913A>T (p.K305*), in the *IL12RB1* gene. Another homozygous variation, c.631C>T (p.R211*), and a frameshift deletion variation, c.315del, in the *IL12RB1* gene were found. One missense variation c.295T>C (p.W99R) and 1 frameshift deletion c.131delC (p. P44fs*) in the *IFNGR1* gene were identified. Twin sisters had the same essential splicing-site variation in intron 6 of *SPPL2A*. Four different monoallelic variations of *STAT1*, c.1492C>G (p.L498V), c.2102A>G (p.Y701C), c.469G>A (p.E157K), and c.2120T>A (p.I707T) were identified in 7 patients. These variations are responsible for AD partial *STAT1* deficiency. Two other brothers had frameshift insertion c.3315_3316insC in the *TYK2* (Table 5).

**Table 5.** Genetic Analysis of Moroccan Patients with Defects in Intrinsic and Innate Immunity

Category	No. of patients	Gene	Transmission	Variation	Protein
MSMD	9	*IL12RB1*	AR	c.913A>T	p.K305*
				c.631C>T	p.R211*
				c.315delG	p. S106Lfs*24
	7	*STAT1*	AD	c.1492C>G	p. L498V
				c.2102A>G	p. Y701C
				c.469G>A	p. E157K
				c.2120T>A	c.2120T>A
	2	*SPPL2A*	AR	c.733+1G>A	Essential splicing site defect
	2	*IFNGR1*	AR	c.295T>C	p. W99R
				c.131delC	p. P44fs*
	2	*TYK2*	AR	c.3315_3316insC	p. T1106Hfs*4
	1	*TBX21*	AR	c.466_471delins AGT TTA	p. E156M157delinsSL
CMC	15	GOF *STAT1*	AD GOF	c.1154C>T	
				c.1154C>T	
				c.820C>T	p. R274W
				c.1885C>T	p. His629Tyr
				c.1633G>A	p. Glu545Lys
				c.265A>T	p. N89Y
				c.511G>A	p. Asp171Asn
				c.812A>C	p. Q271P
				c.800C>T	p. A267V
				c.194A>G	p. Asp65Gly
				c.194A>G	p. Asp65Gly
				c.800C>T	p. A267V
		*IL17RA*	AR	c.596C>A	p. Ser199*
				c.596C>A	p. Ser199*
				c.163+1G>A	
Predisposition to severe viral infection	3	*POLR3A*	AD	c.1909+22g>A	
		*IFIH1*	AD or AR		p. Tyr896*/WT
		*TLR7*	XL		p. Phe690Ile/Y
Predisposition to bacterial infection	3	*IRF4*	AD		p. Phe50Cys/WT
		*IFNGR1*	AD or AR		p. Ser391Gly/WT
		*NCSTN*	AD		p. Ser341Trp/WT
HSE	1	*SNORA31*	AD	n.36 T>C	
IRAK4 deficiency	1	*IRAK4*	AR	c.277deLT	p. F93fsX26

Our study also included 15 patients with CMC (7 male and 8 female). The mean age at onset was 31 months, the mean age at diagnosis was 82 months, and 37% were born to consanguineous parents (Table 1). Fourteen variations have been identified in 2 genes: 3 with AR inheritance in the *IL17RA* gene, and 12 with AD inheritance in the gain of function (GOF) *STAT1* gene (Table 5). Three cases with severe viral infection predisposition were associated with *POLR3A*, *IFIH1*, and *TLR7XL* variations. Additionally, 3 cases of bacterial infection predisposition were linked to variations in *IRF4*, *IFNGR1*, and *NCSTN.* A single case of HSE was identified with *SNORA31* variation, while another case was attributed to *IRAK4* deficiency (Table 5).

## DISCUSSION

Defects in intrinsic and innate immunity are a heterogeneous group of genetic disorders associated with severe and recurrent infections [8, 11]. Although there has been an increase in specialists in clinical immunology, and practitioners' knowledge has grown, which has enhanced early diagnosis and management of this important and rare group of disorders, innate immunodeficiencies still often go undiagnosed. Additionally, the lack of facilities makes diagnosing innate immunodeficiencies particularly challenging in developing countries. It is important to note that delayed diagnosis and misdiagnosis often stem from insufficient knowledge about these conditions among general practitioners and pediatricians [8, 13]. In our cohort, parental consanguinity was present in 51.1% of cases, making it the most predictive factor for defects in intrinsic and innate immunity diagnosis, especially in a disease with an autosomal recessive pattern of inheritance.

The mean value in the delay of diagnosis ranges between 21 and 51 months. This can be due to several factors, including limited diagnostic capabilities at the date of onset or the emergence of some diseases that are new to Moroccan society, which lead to a lack of diagnostic information. From 22 countries in the Middle East and North Africa region, 17,120 patients with IEI have been identified, among whom females represented 39.4%. Parental consanguinity was present in 60.5% of cases. The median age of patients at the onset of disease was 36 months, and the median delay in diagnosis was 41 months [10].

In the current study, a recurrent infection was found in many patients (76%) and was considered the most common clinical manifestation in patients with defects in intrinsic and innate immunity. It is followed by lymphatic and hematologic disorders, pulmonary and respiratory disorders, oral and dental conditions, and dermatologic conditions. The distribution of clinical manifestations in this study closely mirrored that of a study conducted in China, in which respiratory infections, including pneumonia, were identified as the most prevalent complication (79.5%), followed by skin and mucous membrane infections as the second most common (33.9%) [14]. Similar findings were also reported in studies from Egypt [15]. Variations in the prevalence of clinical manifestations across countries could be associated with differences in data collection methods, varying levels of expertise, and the availability of diagnostic facilities. Additionally, the limited accessibility and high cost of medications may have contributed to the increased incidence of these severe complications in our patient population.

In our study, 46 cases were genetically confirmed as 23 were diagnosed with MSMD and 15 were diagnosed with CMC. Additionally, there were 3 cases of severe viral infection predisposition, 3 cases of bacterial infection predisposition, 1 case of HSE, and 1 case of *IRAK4* deficiency. In France, where a larger study was performed, there were 52 patients genetically confirmed as having MSMD, 15 patients confirmed as having HSE, and 97 patients with other innate immunodeficiencies [16]. In Tunisia, there were 21 MSMD cases, similar to our study [17]. In Egypt, there were 6 patients genetically confirmed as having MSMD and 2 patients with other innate immunodeficiencies [18]. Moroccan patients had defects in intrinsic and innate immunity, predominantly MSMD (48%), with genetic variations in the *IL12RB1*, *STAT1*, *IFNGR1*, *SPPL2A*, *TYK2*, and *TBX21* genes [13]. It is followed by CMC (32%) with variations in the *STAT1* and *IL17RA* genes [19]. Our findings, in line with recent research, highlight the involvement of *SNORA31* variations in predisposing individuals to HSE following HSV-1 infection. This variation was also found in a Saudi Arabian patient [20]. For *IRAK4* deficiency, 45 cases have been reported in 13 countries with 20 different variations [21], and this is the first report of the variation c.277deLT p. F93fsX26. We also found that 9 patients (39%) with MSMD had variants in *LL12RB1*, and 7 patients (30%) with MSMD had variants in *STAT1*. In contrast, 12 patients (80%) with CMC had genetic variants in *STAT1* (GOF). This can be considered a distinctive feature of Moroccan community patients, suggesting that cost-effective diagnostic methods, such as PCR, could be used for early detection in this population. In addition to genetic characterization, functional validation studies have provided crucial mechanistic insights into how these variants disrupt immune signaling. For example, *IL12RB1* loss-of-function mutations abolish IL-12 receptor β1 expression, resulting in absent IFN-γ production after IL-12 stimulation, which explains the high burden of disseminated mycobacterial and salmonella infections. Likewise, *IFNGR1* mutations disrupt IFN-γ receptor signaling, resulting in impaired IL-12–dependent macrophage responses. In complete receptor deficiencies (*IFNGR1* or *IFNGR2*), circulating IFN-γ levels are elevated because the receptor is absent from the cell surface and cannot bind or clear the cytokine, rather than as a compensatory increase. *STAT1* variants demonstrate 2 distinct mechanistic consequences: AR loss-of-function mutations abolish *STAT1* phosphorylation or DNA binding in response to IFN-γ, causing MSMD, while AD gain-of-function variants sustain *STAT1* phosphorylation, impair Th17 differentiation, and result in chronic mucocutaneous candidiasis. *TYK2* frameshift mutations reduce, but do not abolish, IFN-γ production after IL-12 stimulation, explaining the incomplete penetrance of mycobacterial and viral susceptibility. Other rare mechanisms include *SPPL2A* deficiency, in which toxic accumulation of CD74 fragments leads to selective cDC2 depletion and reduced Th1 memory responses, and *TBX21* deficiency, which impairs Th1 differentiation and IFN-γ production across lymphocyte subsets [13].

It is still difficult to estimate the prevalence of innate and intrinsic immunodeficiencies in Morocco due to the lack of diagnostic facilities and the high cost of required molecular tests, as innate and intrinsic immunodeficiencies cannot only be diagnosed by cell blood count, immunoglobulins, or lymphocyte subpopulation analysis.

## CONCLUSION

This study focused on the clinical manifestations and genetic variants associated with innate and intrinsic immune disorders. Despite the advances in immunological knowledge and diagnosis, these rare conditions remain underdiagnosed, especially in developing countries with limited resources. Our findings underscore the importance of early genetic diagnosis in improving patient outcomes, as delayed or missed diagnosis can lead to serious complications. The high prevalence of MSMD and CMC in the Moroccan cohort highlights the need for increased awareness among healthcare providers. Furthermore, this study underscores the critical role of genetic variations in these disorders, which, if identified early, can significantly reduce morbidity and mortality. As this research contributes to a broader understanding of innate and intrinsic immune deficiencies, it calls for increased efforts to strengthen diagnostic facilities in Morocco to serve affected populations better.

## AUTHOR CONTRIBUTION

MR, CO, ZA, AE, AM, BB, FA, IB, and AAB contributed to the conception and design of the study. AE, ZA, AM, IB, and AAB gave academic feedback and revised and corrected the manuscript. All authors have reviewed the final manuscript and agreed to be accountable for the work. All authors read and approved the final manuscript.
